# Proteomic Investigation of Aphid Honeydew Reveals an Unexpected Diversity of Proteins

**DOI:** 10.1371/journal.pone.0074656

**Published:** 2013-09-25

**Authors:** Ahmed Sabri, Sophie Vandermoten, Pascal D. Leroy, Eric Haubruge, Thierry Hance, Philippe Thonart, Edwin De Pauw, Frédéric Francis

**Affiliations:** 1 Laboratory of Functional and Evolutionary Entomology, University of Liège, Gembloux, Belgium; 2 Earth and Life Institute, Biodiversity Research Center, Catholic University of Louvain, Louvain-la-Neuve, Belgium; 3 Walloon Center of Industrial Biology, University of Liège, Liège, Belgium; 4 Department of Chemistry – Mass Spectrometry Laboratory, University of Liège, Liège, Belgium; University of Miami, United States of America

## Abstract

Aphids feed on the phloem sap of plants, and are the most common honeydew-producing insects. While aphid honeydew is primarily considered to comprise sugars and amino acids, its protein diversity has yet to be documented. Here, we report on the investigation of the honeydew proteome from the pea aphid *Acyrthosiphon pisum*. Using a two-Dimensional Differential in-Gel Electrophoresis (2D-Dige) approach, more than 140 spots were isolated, demonstrating that aphid honeydew also represents a diverse source of proteins. About 66% of the isolated spots were identified through mass spectrometry analysis, revealing that the protein diversity of aphid honeydew originates from several organisms (i.e. the host aphid and its microbiota, including endosymbiotic bacteria and gut flora). Interestingly, our experiments also allowed to identify some proteins like chaperonin, GroEL and Dnak chaperones, elongation factor Tu (EF-Tu), and flagellin that might act as mediators in the plant-aphid interaction. In addition to providing the first aphid honeydew proteome analysis, we propose to reconsider the importance of this substance, mainly acknowledged to be a waste product, from the aphid ecology perspective.

## Introduction

Insect survival and reproductive success depends on access to balanced carbohydrate and amino acids food sources. This requirement is particularly true in most agricultural monocultures, where nectar and pollen are only available for a short period, or not at all [1]. In such situations, aphid honeydew might be viewed as an alternative food source of key importance to insects, as it contains both plant-derived and aphid-produced sugars and amino acids [2], [3], [4], [5], [6], [7], [8], [9]. In terms of availability, honeydew is the primary and predominant exogenous carbohydrate source in many ecosystems [10]. Available as small droplets or as a thin film on substrates [11], honeydew constitutes a useful food source for many insects (i.e. honeybees, wasps, predatory insects) and vertebrates [4], [12], [13], which consume this aphid excretory product as a source of carbohydrates both for survival and reproduction [14], [15]. However, in comparison to nectar and pollen, honeydew is often viewed as an inferior food source, since it is a waste product [16] that is assumed to only contain a sugar compound matrix.

Aphids feed on the phloem sap of plants [17], [18], [19], and are the most common honeydew producing insects. This excretory product consists of an aqueous mixture of different chemical compounds, with sugars (90–95% of the dry weight) and amino acids being the most important compounds [20]. Many studies have demonstrated that the chemical composition of aphid honeydew varies with (1) host plant species [21], [22], [23], (2) the nutritional state of host plants [24], [25], (3) aphid species, developmental stage, and age [22], [26], [27], , (4) the rate and duration of aphid infestation [30], (5) the presence of ants (mutualism) [31], [32], [33], (6) the presence of bacterial intracellular symbionts [34], (7) parasitism state [35], and (8) the presence of secondary plant metabolites [36]. However, plant-derived phloem sugars (67–89% of the sugar content, including glucose, fructose, sucrose, and maltose) and free amino-acids (78% of the amino acid content, including asparagine, glutamine, glutamate, and serine) seem to be universally present in honeydew [37], [38]. The sugar composition of honeydew reflects the composition of phloem sap; however, a number of other mono-, di-, and oligo-saccharides are also synthesized by the sap feeder (through the action of gut enzymes on plant derived sucrose). Such compounds include melezitose, erlose (fructomaltose), raffinose, and trehalose [1], [16], [22], [25]. The amino acid composition of honeydew corresponds to phloem sap content. Especially, asparagine and glutamine, which are known to dominate in several host plant species used by aphids, were reported as the two major amino acids in honeydew [24], [39].

It is well established that the endosymbiont *Buchnera aphidicola* synthesizes essential amino acids for its aphid host [40]. However, seven non-essential amino acids (glutamate, aspartate, serine, glutamine, alanine, proline and asparagine) are not synthesized by this obligate bacterial symbiont. And, although it was previously suggested that *Buchnera* recycles nitrogenous wastes into essential amino acids, the publication of *Buchnera* genome disproved this hypothesis as neither glutamate dehydrogenase or glutamine synthetase, the two main enzymes for incorporating ammonia, were identified [41]. Nevertheless, a recent transcriptomic analysis provides support for the cooperation of aphid and symbiont gene products in the production of essential amino acids and suggests a possible role of the bacteriocyte (i.e. specialized cells containing the obligatory symbiont *Buchnera*) in recycling ammonia waste for the production of glutamine and glutamate [42].

While aphid honeydew is commonly considered as a source of sugars and amino acids, its importance as a source of proteins has not been previously documented. Here, we report on the first proteomic analysis (2D-PAGE) of honeydew released by a single line of *Acyrthosiphon pisum* (Harris). Supposing that honeydew is composed of proteins from both the aphid host and its harbored bacteria, the identification of honeydew proteins are discussed from the perspective of the producer organisms (i.e. the host aphid or its microbiota).

## Results

**Figure 1:**
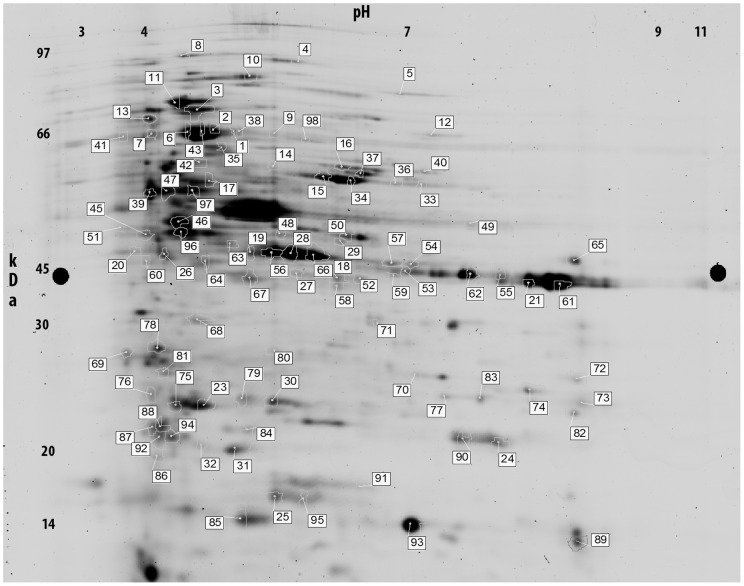
2D-DIGE gel separation of proteins from *Acyrthosiphon pisum* honeydew. Numbered spots corresponded to proteins described in Table 1 and 2.

**Figure 2:**
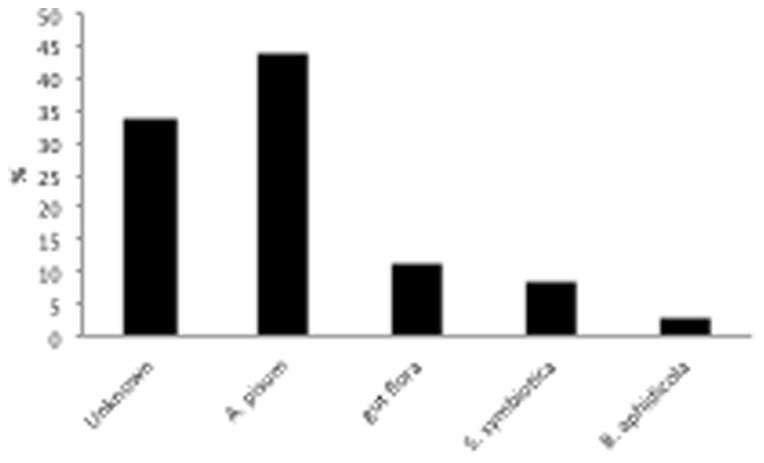
Origin of proteins present in *Acyrthosiphon pisum* honeydew.

While the presence of free amino acids in aphid honeydew has already been described [28], the diversity and abundance of proteins found in the current study was unexpected. Indeed, total protein concentration was high, close to 5 µg/µl suggesting that aphid honeydew might have a nutritional role as source of proteins. A proteomic approach was developed to better characterize the composition of aphid honeydew. More than 140 protein spots were visualized on 2D-PAGE gels (Fig. 1), also represents a diversified source of proteins. To better understand the nature and origin of this unexpected protein diversity, each spot from the 2D gels was analyzed using mass spectrometry. Most of the proteins (67.0%) were identified. A total of 43.8% of proteins corresponded to insect proteins (Table 1), mainly from *A. pisum* (which is actually the only available aphid species sequenced genome). A further 22.7% of proteins originated from bacterial flora (Table 2) associated with the aphid (Fig. 2). The major component of bacterial flora proteins originated from free living bacteria associated with the aphid gut (11.4%) and from secondary symbionts, particularly *Serratia symbiotica* (8.8%). The contribution of the primary aphid symbiont *B. aphidicola* to the honeydew protein composition was relatively low (2.3%).

**Table 1:** Protein identification of *Acyrthosiphon pisum* honeydew separated by 2D-DIGE in the bacteria databases.

Spot number	Protein name	Organism	Code	Nominal mass	pI	PMF		
						Number of peptides identified	Sequence coverage (%)	Mowse score
3	cleft lip and palate transmembrane protein 1	*Acyrthosiphon pisum*	CL194Contig3	45825	10.14	9	17	65
6	Unidentified protein	*Acyrthosiphon pisum*	gi|111159853	28017	9.17	5	15	51
8	splicing factor 3	*Acyrthosiphon pisum*	gi|193664616	85027	5.41	6	21	49
9	adherens junction protein p120	*Acyrthosiphon pisum*	gi|193678745	92494	6.83	6	18	61
13	inorganic pyrophosphatase	*Acyrthosiphon pisum*	gi|328713113	31038	9.59	4	12	58
33	Rap55 protein	*Acyrthosiphon pisum*	gi|193599018	59870	9.39	7	25	59
34	Unidentified protein	*Acyrthosiphon pisum*	gi|46994701	28993	8.07	6	32	52
35	Unidentified protein	*Acyrthosiphon pisum*	gi|158244987	27131	10.08	4	12	52
36	Cullin 1	*Acyrthosiphon pisum*	gi|193610598	90050	8.66	4	13	52
37	Unidentified protein	*Acyrthosiphon pisum*	gi|158232949	22663	10.58	5	29	52
38	Unidentified protein	*Acyrthosiphon pisum*	CL2120Contig2	83434	10.11	12	18	58
39	peroxinectin	*Acyrthosiphon pisum*	gi|193592065	70918	5.46	5	25	50
41	katanin p60 subunit A	*Acyrthosiphon pisum*	gi|193580165	50827	6.49	5	26	56
42	alpha-glucosidase	*Acyrthosiphon pisum*	gi|193620149	76324	6.83	7	23	55
43	Unidentified protein	*Acyrthosiphon pisum*	CL1Contig184	36973	9.53	7	20	56
46	Unidentified protein	*Acyrthosiphon pisum*	gi|158218360	25350	9.92	5	19	50
47	Unidentified protein	*Acyrthosiphon pisum*	gi|158220400	11674	9.33	4	32	54
48	Unidentified protein	*Acyrthosiphon pisum*	CL3866Contig1	52040	10.28	6	13	51
49	Unidentified protein	*Acyrthosiphon pisum*	CL1356Contig2	33717	9.41	8	18	64
50	Unidentified protein	*Acyrthosiphon pisum*	gi|158206451	27671	10.02	5	23	55
51	Unidentified protein	*Acyrthosiphon pisum*	CL10054Contig1	36171	9.45	7	20	59
52	internalin A	*Acyrthosiphon pisum*	gi|193669290	40779	7.63	7	24	58
53	dynamin 1	*Acyrthosiphon pisum*	gi|193697731	78705	5.74	5	56	56
54	peroxidase-like	*Acyrthosiphon pisum*	gi|328720433	46344	6.38	13	31	122
55	Unidentified protein	*Acyrthosiphon pisum*	CL4416Contig1	32061	9.75	7	25	59
56	Unidentified protein	*Acyrthosiphon pisum*	CL6606Contig1	30904	10.07	9	28	71
57	leucyl-tRNA synthetase	*Aedes aegypti*	gi|157113359	134875	6.72	6	22	62
58	Unidentified protein	*Acyrthosiphon pisum*	CL2847Contig1	31938	10.06	7	23	56
59	Unidentified protein	*Acyrthosiphon pisum*	CL460Contig1	56620	10.6	8	14	55
60	Unidentified protein	*Acyrthosiphon pisum*	gi|46998583	47800	3.67	5	23	46
62	electron transport oxidoreductase	*Aedes aegypti*	gi|157137180	34404	8.43	7	22	61
63	transcription initiation factor TFIID	*Acyrthosiphon pisum*	gi|193650189	25722	4.69	8	24	65
64	Unidentified protein	*Acyrthosiphon pisum*	gi|83664017	27381	10.1	7	26	60
66	cop9 complex	*Acyrthosiphon pisum*	gi|193690504	47310	6.85	9	19	55
67	Unidentified protein	*Acyrthosiphon pisum*	CL8202Contig1	26083	9.69	5	21	51
69	RAS-like GTP-binding protein	*Acyrthosiphon pisum*	gi|328713990	32354	8.13	5	16	54
70	2,3-bisphosphoglycerate-independent phosphoglycerate mutase	*Acyrthosiphon pisum*	gi|328725512	74400	10.78	12	15	82
71	Unidentified protein	*Acyrthosiphon pisum*	gi|177779097	27788	9.48	4	14	56
72	myo inositol monophosphatase	*Acyrthosiphon pisum*	gi|193662242	59574	9.11	6	18	62
73	ubiquitin-conjugating enzyme E2M	*Apis mellifera*	gi|48102172	20529	8.21	7	20	66
74	hydroxypyruvate reductase	*Acyrthosiphon pisum*	gi|193659821	36095	8.76	6	23	51
75	Unidentified protein	*Acyrthosiphon pisum*	gi|111158933	33059	9.81	3	13	56
76	alpha-amylase	*Acyrthosiphon pisum*	gi|193669250	64976	5.5	6	18	63
77	Unidentified protein	*Acyrthosiphon pisum*	gi|109194828	27988	10.24	6	20	53
78	rho guanine nucleotide exchange factor	*Acyrthosiphon pisum*	gi|328717372	49666	8.02	6	21	67
80	dihydrofolate reductase	*Drosophila melanogaster*	gi|24647458	20775	6.18	6	47	56
81	fibronectin	*Acyrthosiphon pisum*	gi|193685881	22374	5.13	3	24	52
82	TRAS3 protein	*Acyrthosiphon pisum*	gi|193577747	116462	9.4	6	17	66
83	basic helix-loop-helix protein	*Acyrthosiphon pisum*	gi|193664445	21184	9.33	7	25	54
84	Unidentified protein	*Acyrthosiphon pisum*	CL6375Contig1	28218	9.77	6	24	59
85	Unidentified protein	*Acyrthosiphon pisum*	CL551Contig3	41462	9.57	7	20	55
86	Unidentified protein	*Acyrthosiphon pisum*	gi|158202608	22451	4.34	4	13	46
87	inorganic pyrophosphatase	*Acyrthosiphon pisum*	gi|193720524	15328	4.77	5	11	58
88	Unidentified protein	*Acyrthosiphon pisum*	CL8410Contig1	28766	9.62	7	26	62
89	cathepsin B	*Acyrthosiphon pisum*	gi|161343857	18299	9.24	4	15	57
90	Unidentified protein	*Acyrthosiphon pisum*	gi|158250642	10555	9.91	4	52	51
92	coiled-coil domain-containing protein 75	*Acyrthosiphon pisum*	gi|193697653	29413	4.92	5	17	57
93	Unidentified protein	*Acyrthosiphon pisum*	gi|84647357	15072	10.21	7	35	62
94	prefoldin subunit 5	*Acyrthosiphon pisum*	gi|193713669	17756	4.53	5	21	57
95	Unidentified protein	*Acyrthosiphon pisum*	gi|158220059	27136	9.38	6	18	54

Identification by peptide mass fingerprinting (PMF).

**Table 2:** Protein identification of *Acyrthosiphon pisum* honeydew separated by 2D-DIGE in the arthropod and aphid databases.

Spot number	Protein name	Organism	Code	Nominal mass	pI	PMF		
						Number of peptides identified	Sequence coverage (%)	Mowse score
1	DNA helicase II	*Serratia symbiotica*	ZP_08039051.1	73538	6.22	7	10	59
2	acetyl-coenzyme A synthetase	*Acinetobacter calcoaceticus*	ZP_06058253.1	74468	5.57	11	16	56
4	short-chain dehydrogenase	*Acinetobacter calcoaceticus*	ADY80832.1	77091	5.52	11	21	54
5	histidine kinase	*Escherichia coli*	ZP_07624772.1	74616	5.69	7	10	51
7	phosphoenolpyruvate carboxylase	*Serratia symbiotica*	ZP_08039184.1	62779	5.37	8	14	56
10	aspartyl-tRNA synthetase	*Staphylococcus saprophyticus*	YP_301219.1	68390	6.26	10	18	72
11	chaperone protein DnaK	*Buchnera aphidicola*	NP_777771	70382	5.85	5	13	51
14	Cpn60 chaperonin GroEL	*Serratia symbiotica*	ZP_08039357.1	58111	5.11	7	16	60
15	beta-D-mannosidase	*Staphylococcus saprophyticus*	YP_300188.1	50746	8.74	6	20	52
16	FAD dependent oxidoreductase	*Acinetobacter calcoaceticus*	ADY81259.1	52939	5.98	8	17	50
17	acetyl-coenzyme A synthetase	*Acinetobacter calcoaceticus*	ADY81339.1	57809	6.16	6	11	56
18	transketolase	*Serratia symbiotica*	gi|493760550	72591	5.77	19	14	137
19	pyruvate dehydrogenase	*Serratia symbiotica*	gi|493759916	99586	5.46	30	29	233
21	Cpn60 chaperonin GroEL	*Serratia symbiotica*	ZP_08039357.1	56751	4.86	12	26	111
23	ATP phosphoribosyltransferase	*Acinetobacter calcoaceticus*	ZP_06059232.1	23604	4.80	9	30	81
24	transcriptional regulator	*Acinetobacter calcoaceticus*	ZP_06057412.1	30252	10.72	6	26	82
25	glyceraldehyde-3-phosphate dehydrogenase	*Staphylococcus sciuri*	AAM28576	33623	4.66	11	30	88
26	elongation factor G	*Staphylococcus saprophyticus*	YP_302298.1	57045	5.25	17	26	128
27	chaperone Hsp70	*Serratia symbiotica*	ZP_08040085	69059	4.85	20	32	160
29	chaperone Hsp70	*Serratia symbiotica*	ZP_08040085	45678	5.17	19	12	134
30	ATP synthase beta subunit	*Serratia symbiotica*	gi|493760228	50345	5.00	14	30	133
31	RNA polymerase, beta subunit	*Serratia symbiotica*	gi|493761087	43332	5.23	18	11	120
32	elongation factor Tu	*Serratia symbiotica*	gi|493761094	43368	5.18	19	44	158
40	adenosylmethionine-8-amino-7-oxononanoate aminotransferase	*Buchnera aphidicola*	gi|27904767	48826	9.44	6	22	54
45	tryptophanyl-tRNA synthetase	*Serratia symbiotica*	ZP_08039464	37358	6.16	6	22	66
61	phosphoserine aminotransferase	*Buchnera aphidicola*	gi|15616921	41309	9.41	6	26	60
65	2-isopropylmaltate synthase	*Buchnera aphidicola*	gi|11138482	55910	8.50	9	47	60
68	hypothetical protein SMR0073	*Serratia marcescens*	NP_941147.1	41851	5.39	4	7	57
79	succinyl-CoA synthetase subunit alpha	*Serratia symbiotica*	ZP_08038998.1	30496	5.78	8	35	62
91	uridylate kinase	*Serratia symbiotica*	ZP_08039690	25962	6.10	5	15	52
96	flagellin	*Serratia marcescens*	BAA06987.1	45011	4.90	4	13	38
97	homoserine dehydrogenase	*Acinetobacter calcoaceticus*	ADY83736.1	46933	5.17	6	13	53
98	flagellum-specific ATP synthase	*Escherichia coli*	YP_002329572	49225	5.82	3	7	38

Identification by peptide mass fingerprinting (PMF).

**Figure 3:**
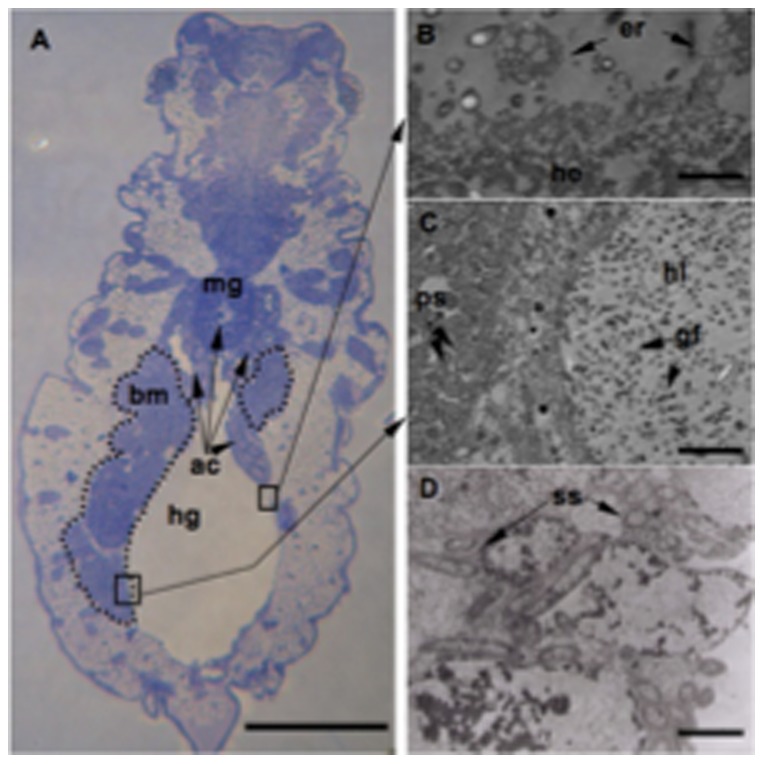
Localization of the bacterial sources of proteins in *Acyrthosiphon pisum* honeydew. **A**. Semithin section of *A. pisum* showing the bacteriome (bm) containing bacteriocytes around the aphid alimentary canal. **B–D**; Transmission electron microscopy micrographs (TEM) of semi-thin sections. In panel (**B**), the ultrastructure of hindgut epithelial cells shows the replacement of old tissues, which are expelled and degraded into the lumen. Panel (**C**) shows the primary symbiont *Buchnera aphidicola* (ps) within a bacteriocyte. Buchnera cells are round and packed into bacteriocyte cytoplasm. The same panel shows that the lumen of the hindgut (hg) appears to be filled with bacteria from gut microbiotae (gf). In panel (**D**), the secondary symbiont *Serratia symbiotica* (ss), which has been indirectly determined by PCR, is enclosed in the cytoplasm of aphid cells in the bacteriome. Scale bars  = 500 µm in A, 3 µm in B, 5 µm in C and 1 µm in D. Abbreviations: ac: Alimentary canal; bm: Bacteriome; hg: Hindgut; mg: Midgut; hl: Hindgut *lumen; ps: Primary* Symbionts; ss: Secondary Symbionts; gf: Gut flora; he: *Hindgut* epithelium; er: epithelium renewal.

Histological analysis confirmed the source of proteins found in *A. pisum* honeydew (Fig. 3A–D). The major source of protein in honeydew originated from the aphid body, appearing to come from tissue renewal. Ultrastructural analysis of the gut confirmed that the hindgut epithelium exhibited dynamic renewal, expelling and degrading tissue into the lumen (Fig. 3B). The gut of *A. pisum* was colonized by a high density of bacterial flora (Fig. 3C), which also contribute some honeydew proteins. The total bacterial flora of honeydew was investigated, and six cultivable bacteria of different prevalence were isolated. All isolates were identified by their 16S ribosomal DNA sequences. The isolates included *Acinetobacter calcoaceticus* (9.10^6^ CFU/ml; Genbank accession no. KC844236), *Staphylococcus sciuri* (3.10^6^ CFU/ml; Genbank accession no. KC844239), *Staphylococcus saprophyticus* (5.10^5^ CFU/ml; Genbank accession no. KC844240), *Serratia marcescens* (2.10^4^ CFU/ml; Genbank accession no. KC905087), *Leucobacter komagatae* (7.10^4^ CFU/ml; Genbank accession no. KC844238), and *Erwinia aphidicola* (3.10^4^ CFU/ml; Genbank accession no. KC844237). The third source of proteins found in aphid honeydew was related to endosymbiotic bacteria. While the aphid primary symbiont *Buchnera aphidicola* was present in bacteriocyte cytoplasm (Fig. 3C), the secondary symbiont, *Serratia symbiotica*, was located in several aphid tissues (including the bacteriome, hemolymph, and gut) (Fig. 3D).

### Aphid proteins

One-third of successful protein identifications with well-known functions were obtained through insect sequence database investigations. Two-thirds of proteins were found to display homology with the pea aphid genome; however, accurate functions were not found. Nevertheless, 19 protein spots were identified as being similar to *A. pisum* aphid protein sequences (Table 1). In addition to several enzymes involved in carbohydrate (α-amylase [spot number 76], phosphoglycerate mutase [spot number 70], and α-glucosidase [spot number 42]) and amino acid (hydroxypyruvate reductase [spot number 74] and cathepsin B [spot number 89]) metabolism of the aphid, two energy related proteins were identified, namely one inorganic pyrophosphatase [spot numbers 13 and 87] and one oxidoreductase [spot number 62]. Several proteins involved in cellular processes were identified in the aphid honeydew; one peroxidase [spot number 54], one inositol monophosphatase (IMPase) [spot number 72] and one dihydrofolate reductase (DHFR) (spot number 80).

### Bacterial proteins

Almost half (16/33) of the identified proteins were homologous with bacterial sequences associated with aphid endosymbiotic bacteria. These sequences were from the primary symbiont *B. aphidicola* [spot numbers 11, 40, 61, and 65] or the secondary symbiont *S. symbiotica* [spot numbers 1, 7, 14, 18, 19, 21, 27, 29, 30, 31, 45, 79, and 91] (Table 2, Fig. 1). Other bacterial proteins were associated to *Staphylococcus sciuri* [spot number 25], *Acinetobacter calcoaceticus* [spots numbers 2, 4, 16, 17, 23, 24, and 97], *Escherichia coli* [spot numbers 5, 32, and 98], *Staphylococcus saprophyticus* [spot numbers 10, 15 and 26], and *Serratia marcescens* [spots number 68 and 96] (Table 2, Fig. 1).

Most of the identified enzymes were involved in amino acid synthesis. These enzymes included one acetyl-coenzyme A synthetase [spot numbers 2 and 17], one ATP phosphoribosyltransferase [spot number 23], one phosphoserine aminotransferase [spot number 61], and one 2-isopropylmalate synthase [spot number 65] for lysine, histidine, serine, and leucine production, respectively. Some other enzymes were related to the citrate cycle; specifically, one phosphoenolpyruvate carboxylase [spot number 7] and one pyruvate dehydrogenase [spot number 19]. A short-chain alcohol dehydrogenase [spot number 4] and a signal transduction histidine kinase [spot number 5] were also identified, which are also involved in energy metabolism. In addition succinyl-CoA synthetase [spot number 79] was identified, which is the only mitochondrial enzyme capable of ATP production via substrate level phosphorylation without oxygen, in addition to playing a key role in the citric acid cycle. Some of the identified proteins were shown to be involved in the response of plants to bioagressors, including several chaperones from *B. aphidicola* [spot number 11] and *S. symbiotica* [spot numbers 14, 21, 27, and 29]. The major chaperone systems of bacterial cells were identified in aphid honeydew; including, GroEL [spot numbers 14 and 21], DnaK [spot number 11], and Hsp70 [spot numbers 27 and 29] chaperones. Another well-known elicitor of plant defense, flagellin (flg) [spot number 96] from *S. marcescens*, was also found in *A. pisum* honeydew. Finally, some elongation factors from *S. saprophyticus* and *E. coli* [spot numbers 26 and 32] were also identified.

## Discussion

To date, aphid honeydew is considered as primarily comprised of carbohydrates. Although the experiments reported here have been executed on a single aphid line and thereby deserve to be repeated on additional aphid lines and species, our results provide new insights into a substance previously considered as a waste product.

First, the current proteomic analysis (2D-PAGE) of *A. pisum* honeydew allowed the isolation of more than 140 protein spots, demonstrating that aphid honeydew represents a diverse source of proteins. Interestingly, our results reveal that the protein diversity of aphid honeydew originates from several partners (i.e. the host aphid and its microbiota, including endosymbiotic bacteria and gut flora). Indeed, 60 spots matched to insect database sequence resources, while 36 spots were identified to be homologous to bacterial sequences. Almost half of the bacterial identified proteins were homologous to bacterial sequences associated with aphid endosymbiotic bacteria. Most of the bacterial proteins identified in honeydew (27.8%) were related to the genetic information process, while 20% of the bacterial symbiont proteins were related to the amino acid metabolism.

Second, the current proteomic approach allowed the identification of some proteins that might act as mediators in the plant-aphid interaction. Indeed, the proteins flagellin [spot number 96] and elongation factor Tu [spot number 32], identified from the pea aphid honeydew, are known to act as inducers of defenses in many plant species [43], [44], [45]. Flagellin (flg) is the main building unit of the eubacterial flagella while the elongation factor Tu (EF-Tu) is the most abundant protein in a growing bacterial cell [46]. Most plant species (tomato, tobacco, potato and *Arabidopsis* suspension cultures) respond to a conserved 22-amino-acid epitope, flg22, present at the flagellin N-terminus [47] and the N-terminal 18 amino acids of EF-Tu (elf18) triggers plant basal defenses [45], [46]. Beside its primary role in protein synthesis, bacterial elongation factor Tu (EF-Tu) was found to induce defensive responses in plants, mainly in Brassicaceae such as bacterial resistance in *Arabidopsis thaliana* to *Pseudomonas syringae* bacterial plant pathogen [48]. Major chaperone systems in bacterial cells, GroEL [spot number 21], DnaK [spots number 11] and Hsp70 [spot number 27 et 29] were found in aphid honeydew. Molecular chaperones assist the protein folding in the cell but are also involved in numerous processes in bacterial cells, including assisting the folding of newly synthesized proteins, both during and after translation; assisting in protein secretion, preventing aggregation of proteins on heat shock, and repairing proteins that have been damaged or misfolded by stresses such as heat shock [49]. Although their role in plant defense is not well described, molecular chaperones have been reported to be components of the hypersensitive response in *Nicotiana benthamiana* or to facilitate associations of multiple proteins involved in pathogen recognition [50]. Chaperonin [spot number 14] from *Buchnera* was found to be a major protein in the hemolymph of several aphid species including *A. pisum* [51]. However, it should be noted that the role of aphid honeydew in elicitation of plant defense responses has not been demonstrated yet, nevertheless, in light of our results, this deserves to be investigated.

Finally, the current study also raise to question of the nutritional value of aphid honeydew as well as its role from a multitrophic perspective. In natural ecosystems, aphids provide an important link in the food chain. They serve as a food source for many insect predators, and are essential for the successful reproduction of several parasitoids [52]. The aphid honeydew might also contribute to the local biodiversity by attracting some pollinators such as syrphids. Indeed, it has been recently demonstrated that some aphidophagous species (i.e. syrphids and ladybirds) use aphid honeydew to locate their aphid prey. However, to date, aphid honeydew has never been considered as an alternative food source because its excretory product is considered of poor nutritional quality [16] compared to nectar and pollen [24], [37], [38], [39]. On the contrary, we report an unexpected diversity of proteins in aphid honeydew, which has not been previously recorded in the published literature. Therefore, the protein content of aphid honeydew might represent a valuable food source for herbivorous insects, by providing a combination of sugar, amino acids, and proteins. Indeed, plants covered by honeydew have been observed to attract a multitude of flying and crawling insects; thus, promoting high biodiversity in their immediate environment (Francis, personal communication).

Honeydew is also the keystone on which ant-aphid mutualism is built. To date the mutualistic interaction between aphids and ants was only studied from the perspective of the sugar composition of aphid honeydew, and the use of carbohydrates by aphid-associated entomofauna. However, some studies suggested that the ratio of carbohydrate and protein resources available to ants influence their decision to participate in the mutualism and the longevity of the colony [53], [54]. Thus, considering the proteins/carbohydrates balanced profile of aphid honeydew might be of interest in order to gain a more general understanding of how aphid honeydew might guide ant-aphid interactions.

In conclusion, in addition to provide the first analysis of the aphid honeydew proteome, the current work invites to not consider it as a simple waste product and suggests to investigate its nutritional role as well as its potential implications in multitrophic interactions.

## Materials and Methods

### Biological material

In a climate-controlled room (16 hr light photoperiod; 60–70% RH; 20±2°C), the host plants, *Vicia faba* L. (var. Major), were grown in 9×8 cm plastic pots containing a mixture of vermiculite and perlite (1/1), and were infested with the aphid *Acyrthosiphon pisum* Harris. This aphid species was collected from field crops in 1990, and has been reared for years at the University of Liege, Gembloux Agro-Bio Tech (Department of Functional and Evolutionary Entomology), Belgium. Aphids are transferred onto new *V. faba* host plants once a week, and maintained in the same climate-controlled room.

### Honeydew collection and conditioning

The collection of aphid honeydew was carried out under aspectic conditions in a laminar flow hood and observing proper handling procedure. Several *V. faba* plants that were heavily infested with the aphid *A. pisum* were placed 10 cm above a sterile aluminum foil. Using sterile microcapillaries of 10 µl volume, only honeydew droplets that fell onto the aluminum sheet were directly collected as samples of freshly produced honeydew. Honeydew droplets remaining on leaves were not collected in order to prevent contamnination by the phyllosphere.

### Identification of honeydew and aphid bacterial contents

To investigate the microflora of honeydew, 100 µl of *A. pisum* honeydew was collected as described above. A series of ten-fold dilutions was made into a saline solution (containing per liter of distilled water, 0.9 g of NaCl, 1 g of casein peptone and 1 g of tween 80). Then, 100 µl of each dilution was plated on 868 agar medium (containing per liter of distilled water, 1.7% of agar and 10 g of glucose, yeast extract, and casein peptone). Colonies were visible after 24 to 48 h of incubation at 25°C, and the strains were then isolated and purified on the same medium.

For bacterial identification, genomic DNA was extracted from cells grown at 25°C for 48 h, and PCR amplification of the 16S ribosomal DNA sequences was performed. Genomic DNA was purified by using the Wizard Genomic DNA purification Kit (Promega). The primers used for PCR amplification of 16S ribosomal DNA sequences were the universal primers 16SP0 (5′-GAAGAGTTTGATCCTGGCTCAG-3′) and 16SP6 (5′-CTACGGCTACCTTGTTACGA-3′). The PCR mixture contained PCR Buffer, 2 mM MgCl_2_, 1 U of Taq polymerase (Fermentas), and dNTP at a concentration of 20 mM (Promega). The running parameters were 25 cycles of 95°C for 30 s, 55°C for 30 s, and 72°C for 2 min; the denaturing step was 5 min and the final extension was 10 min. The PCR product was purified using GFX PCR DNA and a Gel Band Kit (GE Healthcare), then sequenced using Big Dye v3.1 Kit and 3730 DNA Analyser (Applied Biosystems). The obtained sequences (400–600 bp) were assembled with the program BioEdit 7.1.9. Although no new sequence data was generated, all new data has been deposited in GenBank.

The secondary symbionts harbored by the *A. pisum* clone were checked by diagnostic PCR analysis using the specific primer sets listed by [55]. Five known secondary symbionts of *A. pisum* (PASS, PAUS, PABS, *Rickettsia* and *Spiroplasma*) and two facultative endosymbionts found in various insects (*Wolbachia* and *Arsenophonus*) were targeted.

### 2D polyacrylamide gel electrophoresis

Proteins from fresh honeydew were precipitated using the 2D Clean Up Kit according to the manufacturer's instructions (GE Healthcare), and resuspended in a 7 M urea, 2 M thiourea 20 mM Tris pH 8.5 buffer, which contained 1% CHAPS and 1% ASB14. Quantification of the precipitated proteins was realized using the RCDC quantification kit from Bio-Rad. The protein extract (samples of 25 µg) was labeled with one of three CyDyes (GE Healthcare), following the standard DIGE protocol, and was adjusted to a volume of 450 µl, which was used to rehydrate 24 cm IPG strips (pH 3–10 NL from GE Healthcare) for 12 h at 20°C, and a constant voltage of 50 V. Isoelectric focusing (IEF) was carried out at 200 V for 200 Vh, 500 V for 500 Vh, 1000 V for 1000 Vh, and 8000 V for 60000 Vh at 20°C, and a maximum current setting of 50 µA/strip in an isoelectric focusing unit from GE Healthcare. Following IEF, the IPG strips were equilibrated for 15 min in 375 mM Tris (pH 8.8), containing 6 M urea, 20% v/v glycerol, 2% w/v SDS, and 130 mM DTT, and were then kept for a further 15 min in the same buffer, except that DTT was replaced with 135 mM iodoacetamide. The IPG strips were then sealed with 0.5% agarose in SDS running buffer, at the top of gels polymerized from 12% w/v acrylamide and 0.1% N,N'-methylenebisacrylamide. Second-dimensional electrophoresis was performed at 20°C in an Ettan Dalt-six electrophoresis unit (GE Healthcare) at 25 W/gel for 5 h. Gels were scanned with a Typhoon fluorescence imager (Amersham), at wavelengths corresponding to each CyDye. Images were analyzed with SameSpots software version 3.2 (Non Linear Ltd, Newcastle) according to the manufacturer's instructions. Gels were completed in three replicates.

### Proteins identification

A non-labeled 300 µg sample of aphid honeydew protein was added to one of the analytical gels, and the protein spots were excised from the gel using an Ettan spotpicker robot (GE Healthcare). Selected gel pieces were collected in 96-well plates designed for the Perking Elmer automated digester. Briefly, gels pieces were washed with 3 alternative soakings in 100% ammonium hydrogenocarbonate 50 mM and a mix of 50% Acetonitrile and 50% ammonium hydrogenocarbonate 50 mM. Two additional washes were performed with 100% acetonitrile to dehydrate the gel. A volume of 3 µl of freshly activated trypsin (Roche, porcine, proteomics grade) 10 ng/µl in ammonium hydrogenocarbonate was used to rehydrate the gel pieces at 8°C for 30 min. Trypsin digestion was performed for 3 h at 30°C. Peptide extraction was performed with 10 µl of 1% formic acid for 30 min at 20°C.

Protein digests (3 µl) were adsorbed for 3 min on prespotted anchorchips (R) using the Perkin Elmer robot. Spots were washed “on-target” using 10 mM dihydrogeno-ammonium phosphate in 0.1% TFA-MilliQ water to remove salts. High throughput spectra acquisition was performed using an Ultraflex II MALDI mass spectrometer (Bruker) in positive reflectron mode, with close calibration enabled, the Smartbeam laser focus was set to medium, and a laser fluency setting of 65 to 72% of the maximum was used. Delayed extraction was set to 30 ns. Steps of 100 spectra in the range of 860–3800 Da were acquired at a 200 Hz LASER shot frequency, with automated evaluation of intensity, resolution, and mass range. A total of 600 successful spectra per sample were summed, treated, and de-isotoped in line with an automated SNAP algorithm using Flex Analysis 2.4 software (Bruker). The samples were then submitted in the batch mode of the Biotools 3.0 software suite (Bruker), with an in-house hosted Mascot search engine [56] (MatrixScience.com) connected to the NCBI non redundant database with parameters set for Metazoa and Bacteria. Specific searches toward *Buchnera – Serratia – Acyrthosiphon pisum* aphid databases were also performed. A mass tolerance of 80 ppm with close calibration and one missing cleavage site was allowed. Partial oxidation of methionine residues and the complete carbamylation of cystein residues were considered. The probability score calculated by the software was used as one criterion for correct identification. Experimental and Mascot results of molecular weights and pI were also compared.

To categorize the identified proteins based on metabolic function, searches were performed using the Kegg pathway database (http://www.genome.jp/kegg/pathway.html) and Expasy Proteomic tools (http://www.expasy.org/tools/), particularly the Biochemical–Metabolic pathway sections.

### Histological analyses

Semi-thin and thin sections were performed. Aphids were fixed by direct immersion for 3 h at room temperature in a 2.5% glutaraldehyde solution, buffered with 0.2 M Na-cacodylate at pH 7.4. The osmolarity was adjusted to 850 mOsm by the addition of sucrose (5%). All samples were post-fixed in glutaraldehyde for 2 h at 4°C in buffered 1% OsO 4, rinsed in distilled water, dehydrated in an ethanol-propylene oxide series, and embedded in epoxide (Glycidether 100, Serva). Flat silicone rubber molds were used to facilitate orientation before sectioning. Aphids were cut into several semi-thin sections (1 μm thick) using glass knives (Ultramicrotome LKB or Reichert-Jung Ultracut E). The sections were then stained with toluidine blue for light microscopy in 1% toluidine blue at pH 9.0 before observation under an Olympus microscope. Selected samples were cut into ultra-thin sections for transmission electron microscopy with a diamond knife and contrasted with uranyl acetate and lead citrate before examination with a JEOL TEM (JEM 100-SX) at 80 kV accelerating voltage.
